# Weekly low-dose methotrexate for reduction of Global Initiative for Asthma Step 5 treatment in severe refractory asthma: study protocol for a randomized controlled trial

**DOI:** 10.1186/1745-6215-15-492

**Published:** 2014-12-18

**Authors:** Riccardo Polosa, Salvatore Bellinvia, Massimo Caruso, Rosalia Emma, Angela Alamo, Marek Leszek Kowalski, Christian Domingo

**Affiliations:** Department of Clinical and Biomolecular Medicine, University of Catania, Ospedale Garibaldi Nesima, 636 Via Palermo, 95122 Catania, Italy; Internal and Emergency Medicine, Policlinico - V. Emanuele, University of Catania, 78 Via Santa Sofia, 95123 Catania, Italy; Department of Immunology, Rheumatology and Allergy, Medical University of Lodz, 251 Pomorska Str. 92-213, Łódź, Poland; Department of Medicine, Autonomous University of Barcelona, Servei de Pneumologia Corporació Sanitària Parc Taulí, Parc Taulí 1, 08208 Sabadell, Barcelona, Spain

**Keywords:** Chronic severe asthma, Immunomodulation, Methotrexate, Steroids, Omalizumab, Randomized controlled trial

## Abstract

**Background:**

Patients with chronic severe asthma (CSA) have a crippling disease and current available treatments are not satisfactory. Thus, management of CSA remains a major unmet need. Although the evidence from existing randomized controlled trials fails to support a definite role for immunomodulatory drugs in these patients due to major methodologic drawbacks, findings with low-dose methotrexate (MTX) are encouraging. However, larger and well-designed clinical trials are required to establish the beneficial role of MTX in CSA, and for the detection of the key characteristics of those who are going to respond to this drug.

**Methods/design:**

Patients will be recruited from the accessible asthmatic patients lists of tertiary referral centers. All patients will meet the stringent diagnostic criteria for CSA, including the requirement for the regular use of Global Initiative for Asthma (GINA) Global Strategy for Asthma Management and Prevention Step 5 medications (oral prednisone and/or omalizumab). The experimental design of the proposed study will take the form of a double-blind parallel-randomized placebo-controlled trial consisting of a total of eight visits, including run-in and run-out periods. Patients will be randomly allocated to receive either MTX or a matched placebo once a week as an add-on therapy to their existing medication after run-in. Physiological, laboratory and clinical assessments will be measured regularly throughout the study and compared with baseline assessments.

**Discussion:**

We expect that MTX will reduce Step 5 medications dosage in patients with CSA without compromising the overall disease control. Improvement in several indicators of asthma severity and control will be also investigated.

**Trial registration:**

ClinicalTrials.gov Identifier: NCT02124226 (assigned 25 April 2014).

## Background

Asthma is a multifactorial disease characterized by various phenotypes and heterogeneous pathological mechanisms, which affects 5 to 20% of the population in Europe, North America and Australia with a rising prevalence, particularly amongst pediatric and elderly populations [1–3].

Over the last 50 years, there has been an escalation in knowledge about the cells and mediators involved in the disease’s pathogenesis. The identification of the Th2 cell as the key ‘orchestrator’ of the allergic response, culminating in the interleukin (IL)-4 and IL-13 dependent generation of immunoglobulin E (IgE) by dedicated follicular B cells and plasma cells, has represented a central finding for many years [4]. However, alternative T-cell subtypes (for example, Th1 cells, regulatory T-cells and Th17 cells) are now being taken into consideration [5], and mixed endotypes are currently being proposed [6].

Owing to the complex nature of the disease, it is not surprising that many asthma patients continue to have uncontrolled disease that requires either improved adherence to medications or high intensity treatment [7–9]. Patients with severe unremitting disease have the greatest impairment of their quality of life and account for a disproportionate use of healthcare resources through hospital admissions, unscheduled doctor’s visits and use of emergency services [10, 11]. The accurate prevalence of such cases is unknown, but may fluctuate around 5 to 8% of the total asthma population, depending on the definition [12, 13].

Asthmatic patients with this aggressive phenotype respond poorly to standard treatments and acceptable control of the symptoms can only be achieved by taking regular systemic corticosteroids [14, 15]. Although the lowest possible dose should be prescribed, this subgroup of patients often needs high doses of oral corticosteroids (OCS) (prednisone ≥25 mg/day) in order to attain an adequate asthma control and exhibits a deterioration as soon as the dose of corticosteroids is tapered. Furthermore, some of these patients may respond poorly to oral prednisone because of an intrinsic form of insensitivity to corticosteroids [16]. Hence, reasonable control of their asthma can then only still be achieved at the cost of significant adverse effects.

The past years have witnessed the successful introduction of a new drug entity for the severe asthma phenotype: the monoclonal anti-IgE antibody, omalizumab. Nonetheless, omalizumab is currently approved for add-on use only in the small subset of allergic severe asthma patients [17, 18]. Therefore, for non-allergic asthma patients with severe chronic disease, a combination of OCS with a variety of oral and nebulized bronchodilators, in addition to high-dose inhaled corticosteroids and beta2 agonists, is the only recommended strategy by current international asthma guidelines. Collectively, omalizumab and systemic steroids represent the most intensive level of treatment schedule for asthma and constitute the so-called treatment category ‘Step 5’ in the Global Initiative for Asthma (GINA) Global Strategy for Asthma Management and Prevention guidelines [18].

It is clear, that the current therapeutic arsenal is unsatisfactory and management of asthmatic patients with severe refractory disease remains a major unmet need. Despite the development of a plethora of new biologic therapies targeting specific inflammatory cells or receptors of inflammatory mediators relevant to asthma, these highly specific tools can achieve only limited improvement in patients with severe refractory disease and are very expensive [19, 20].

Clinical experience with immunomodulatory agents for the treatment of patients with chronic inflammatory rheumatic diseases suggests that these therapies are very effective and at low dose appear to be safe for long-term use [21]. The notion that the efficacy of these agents is also resulting from their well-known steroid-sparing activity can be exploited in other inflammatory diseases, including chronic severe asthma. Therefore, a more widespread use of current immunomodulatory therapies for chronic severe asthma has been proposed [22, 23]. Specifically, weekly low-dose methotrexate (MTX) has been shown to substantially decrease daily prednisone dose in steroid-dependent asthmatic patients in randomized controlled trials [24].

Large and well-designed controlled studies are now required to establish the role of MTX in chronic severe asthma patients and to detect the key characteristics of those who are going to respond to this drug. With this in mind, we designed a prospective randomized controlled trial in well-characterized patients with chronic severe asthma to evaluate the efficacy of an add-on weekly low dose of MTX in reducing the total dose of Step 5 medications (OCS and/or omalizumab) without deterioration in respiratory symptoms and airway function. We will also monitor low-dose MTX adverse events and tolerability.

## Methods/Design

### Participants

Patients with chronic severe asthma will be recruited from the accessible asthmatic patients lists of tertiary referral centers to ensure the recruitment of 102 patients. All patients will meet the current GINA diagnostic criteria for chronic severe asthma, which includes the requirement for the use of GINA Step 5 medications (oral prednisone and/or omalizumab) [18]. Full details of antiasthma treatment use including high-dose inhaled corticosteroids, leukotriene modifiers, theophylline, long acting beta-agonists, long acting muscarinic antagonists, will be recorded at enrolment.

#### Inclusion criteria

Study inclusion criteria are as follows:

1. patients with a diagnosis of chronic severe asthma taking GINA Step 5 medications (regular OCS and/or omalizumab for a minimum of six months);
2. failure in weaning patients completely from Step 5 medications during run-in;
3. male and female individuals aged 18 to 75 years;
4. patients must be able to provide consent.

#### Exclusion criteria

Study exclusion criteria are as follows:

1. use of immunomodulatory therapies in the preceding three months;
2. recent or current history of alcoholism;
3. high liver enzyme levels (greater than 2.5 times the upper limit of the normal range);
4. serum creatinine levels greater than 2.0 mg/dL;
5. acute illness within 15 days of study medication administration;
6. leucopenia (below 3.0 × 10^9^/L) and/or thrombocytopenia (below 100 × 10^9^/L);
7. pregnancy or planning to become pregnant;
8. evidence of pulmonary fibrosis or chronic liver disease.

Patients thought to have uncontrolled asthma as a consequence of co-existent conditions (such as gastro-esophageal reflux or chronic rhinosinusitis) will not be excluded. Participants will be allowed to adjust controller and reliever medication use as necessary throughout the study and these details will be reported in a clinical diary. The physician in charge will explain the objectives of the study and obtain informed consent from patients invited for the enrolment visit. Patient organizations will also locally assist the enrolment procedure by informing their patients about the study. We anticipate to complete enrolment within six months. The study protocol has been approved by the Ethics Committee of the Azienda Ospedaliera Universitaria Policlinico - V Emanuele di Catania (record number: 682; 28-01-2014) in accordance with the Helsinki Declaration. The coordinating centre has centralized ethical approval. Ethical approval from each participating centre will be obtained prior to patients’ recruitment.

### Trial design

The design is a double-blind parallel-randomized placebo-controlled trial. The placebo and the active drug will be provided by the study sponsor; the company will not be involved in the study design, data collection, analysis or interpretation of the data. According to sample size estimation, a total of 102 patients will enter the study (see Sample size estimates section below).

**Figure 1:**
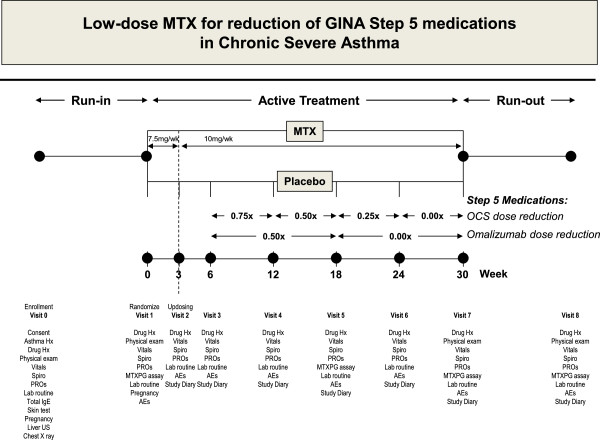
**A schematic diagram of the study design.** MTX: Methotrexate. PROs: Patient Reported Outcomes. US: Ultrasound. MTXPG: Methotrexate Polyglutamate. AEs: Adverse Events.

A schematic diagram of the study design is presented in Figure 1. In brief, patients will be invited for an enrolment visit (visit 0) to check for eligibility and for baseline measurements. This visit will be followed by a run-in period during which optimization of OCS doses prior to randomization will be achieved. Steroid doses will be progressively tapered (5 mg/day of prednisone every week) until a decrease of >10% in the forced expiratory volume in 1 second (FEV1) is observed. Steroid dose will then be increased to the previous level and the tapering strategy will be repeated once again to establish the minimum dose needed to stabilize FEV1. Patients on omalizumab will be also subjected to a dose-reduction strategy; doses will be progressively reduced by 50% every six weeks until a decrease of >10% in the FEV1 is observed. If necessary, the omalizumab dose will then be increased to the previous level.

At the end of the run-in period, patients will attend an entry visit (visit one) to confirm eligibility and to repeat baseline measurements. At this time, patients will be randomly allocated to receive either MTX (starting dose of 7.5 mg/week plus folic acid the day after) or matched placebo (once a week for three weeks) as an add-on therapy to their existing medication. Randomization will be undertaken by a professional contract research organization (CRO). At the subsequent visit (visit two) three weeks later, study treatment dosage will be increased (thus, for those on MTX, the maintenance dose will be 10 mg/week plus folic acid the day after). The maintenance dose will be administered for a total of 27 weeks. Selection of the dose and duration of treatment are chosen from seminal clinical trials of MTX in patients with active rheumatoid arthritis [21]. Progressive stepwise dose-reduction in GINA Step 5 medications will begin three weeks later, at visit three. If possible, dose reductions will be attempted every six weeks for OCS and every 12 weeks for omalizumab, according to the strategy illustrated in Figure 1. Physiological, laboratory and clinical assessments will be repeated regularly at six-week intervals (visits four to seven).

At the end of the active treatment period (visit seven), patients will enter a run-out period, during which modifications of the patients’ asthma symptoms and medications will be carefully documented. At the end of the run-out period, patients will attend a final visit (visit eight) to repeat physiological, laboratory and clinical assessments. At visits five, seven and eight a blood sample will be taken for estimation of intracellular levels of MTX polyglutamates (MTXPGs) in blood cells for possible clinical correlations and prediction of MTX responders. MTXPGs assay will be carried out centrally.

The proposed timetable for conducting and completing the study is summarized below:

1. Consensus agreement on diagnostic criteria, standardization of procedures, investigators training sessions and ethical approvals: three to four months;
2. Recruitment phase and patients inclusion: six to eight months;
3. Run-in phase and verification of inclusion criteria: three to four months;
4. Randomization followed by treatment phase: seven months;
5. Run-out phase: 12 months;
6. Data collection, data check and statistical analysis: three to four months;
7. Final report: one month.

### Study intervention

Patients randomly allocated to receive MTX will be given a subcutaneous injection once a week (initially 7.5 mg/week for three consecutive weeks, followed by a maintenance dose of 10 mg/week for a total of 27 weeks) as add-on therapy to their existing medication. The placebo is matched to have an identical appearance to the active drug. A prescription of 5 mg folic acid per week with MTX therapy is recommended to reduce side effects; folic acid will be taken by all participants (both MTX and placebo-treated). Treatment dose and duration are chosen from seminal clinical trials of MTX in patients with active rheumatoid arthritis [21]; MTX has a sound safety profile according to the evidence from these trials and the longstanding experience with its use. If lab results are suggestive of MTX toxicity, treatment will be suspended and the patient will be withdrawn from the study, with re-evaluation after three to four weeks. Regarding less severe side effects, such as stomatitis and/or abdominal discomfort, these respond well to symptomatic treatment. The active drug and matched placebo will be provided by the study sponsor.

### Study outcomes

#### Primary outcome measures

According to the most recent revision of the international asthma guidelines [18], the combination of OCS and/or omalizumab in addition to high-dose inhaled corticosteroids and bronchodilators to achieve control (falling under the highest category known as Step 5 medications) is recommended for patients with chronic severe asthma. Therefore, the primary measured outcome of efficacy for this study will be: at least 50% reduction in total dosage of Step 5 medications (systemic steroids and/or omalizumab) without compromising the overall disease control. Dose reductions from baseline can be calculated from the clinical diary. Reductions in the total dose of Step 5 medications by MTX will reduce the risk of serious adverse effects and will lead to considerable cost savings.

#### Secondary outcome measures

All major clinical trials on patients with chronic severe asthma have substantially demonstrated that FEV1 is not a realistic clinical endpoint for this disease phenotype [25–28]. For this reason, we considered it inadequate to accept FEV1 as a primary endpoint in this study proposal. Alternatively, for some of these clinical trials, patient-reported outcomes such as the Asthma Quality of Life Questionnaire (AQLQ) and the Asthma Control Questionnaire (ACQ) have proved to be acceptable clinical endpoints [29, 30]. Therefore, the secondary measured outcomes of efficacy for this study will be:

1. no significant deterioration in lung function (FEV1);
2. a clinically significant change (at least 0.5 point) in the AQLQ score [29];
3. a clinically significant change (at least 0.5 point) in the ACQ score [30].

The number of severe asthma exacerbations from the previous follow-up visit (a severe asthma exacerbation is defined as an increase in respiratory symptoms requiring a short course of oral or parenteral corticosteroids) will be also recorded throughout the study and used as additional secondary measured outcomes of efficacy. Dose reductions in inhalational therapy from baseline will also be calculated from the clinical diary. Incidence of hepatotoxicity will be used as safety outcome.

### Methotrexate polyglutamates assay

Samples of heparinized peripheral whole blood (5 mL) obtained from participants receiving MTX will be centrifuged at a low speed for 10 minutes to pellet the red blood cells (RBCs). RBCs are then resuspended in an equal volume of normal saline (Baxter Deerfield, IL, USA), mixed by gentle inversion, and subjected to a second low-speed centrifugation. After discarding the supernatant, the packed RBCs are divided into two aliquots and stored at −70°C until analysis. To increase specificity and minimize interference with coeluting substances, the ion-pair reverse-phase chromatography (IPRPC) method is used to identify methotrexate glutamate (MTXGlu) in human RBCs to profile mono- and polyglutamated folates. Briefly, sample preparation consists of a simple protein-precipitation step, followed by solid-phase extraction. The reconstituted sample is separated by IPRPC with N, N-dimethylhexylamine as an ion-pairing reagent (Sigma-Aldrich Corp., St. Louis, MO, USA), resulting in an elution order proportional to the MTX polyglutamation status. Detection of each MTXGlu is carried out using a micromass Ultima triple quadropole mass spectrometer (Agilent Technologies, CA, USA), operating in positive ion mode. The lower limit of detection is found to be in the 0.1 to 0.5 nM range for each MTXGlu.

### Blinding and randomization

In this parallel two-arm double-blind, placebo-controlled study, patients with chronic severe asthma taking GINA Step 5 medications will be randomized to either MTX or matched placebo. The randomization sequence will be generated by an *ad-hoc* statistical program. A statistician will run the program to generate a random list on the basis of the number of patients and the treatment blocks. Once printed, the randomization list will be sealed in a close enveloped and archived. Masking of the active drug will be professionally carried out by a professional CRO; the personnel involved in administering interventions and assessing outcomes at the participating centers will not be aware of treatment assignment.

### Information retrieval

Patients’ data will be collected by means of an electronic case report form (eCRF). The eCRF is built up on the basis of the study protocol and is divided into visits sections on the basis of the study design. Prior to its implementation into the study, eCRF will be approved for adherence with current legislation for privacy and data protection. Data collected with eCRFs will be automatically feeding a central study database. An eCRF training session about data entry procedures will be provided to all those involved. Potential data entry errors that could be made by the medical staff at the site will be reduced by data verification activities by the CRO in charge, and resolved either through monitoring visits or through *ad-hoc* teleconferences.

### Organizational aspects and study feasibility

Recruiting centers will be selected on the basis of their expertise in the management of chronic severe asthma, availability of a significant number of these patients’ phenotype and documented experience in clinical trials. Working definitions for this study have been already discussed, reviewed and agreed, together with the analysis of potential recruitment impediments. Moreover, focusing on sound and clinically relevant primary outcome measures it is likely to improve the scientific yield and optimize overall recruitment. A competitive recruitment strategy will be employed to ensure optimal patients are recruited in a timely manner.

Coordination and management will be provided by a professional CRO. Before the commencing the study, investigators will be invited to a kick-off meeting during which consensus agreement on diagnostic criteria will be reached, the study protocol will be explained in detail and a specific training session about the standardization of all study procedures, the treatment scheme and good clinical practices (GCP) will be offered. The CRO will be in charge of training sessions and the monitoring of GCP principles throughout the study. The CRO will monitor the quality of the data and the progress of the study, with an eye towards identifying delays early and working with investigators to develop rapid solutions. A list of additional clinical centers is already available for replacement in case of the study underperforming. MTX-PG assay will be carried out centrally. Statistical analyses of the data and realistic delivery of the results is guaranteed by the involvement of a dedicated team of biostatisticians.

### Sample size estimates and statistical analysis

A total of 102 patients will enter this two-treatment parallel-design study. The sample size is calculated to give 90% power to reject a two-sided test of the null hypothesis that there is no treatment difference between MTX and placebo, with respect to the primary outcome variable. The probability is that the study will detect a treatment difference at a two sided 5.0% significance level if the true difference between the treatments is 4.56 units. This is based on the assumption that the standard deviation of the response variable is 5.1 [24]. The requisite for stratification by daily (high or low) prednisone dose at randomization and a dropout estimate of 20% of the participants have been also taken into account.

Our statistical hypothesis is that the study will be able to show the superiority of MTX treatment versus placebo so that it significantly reduces the individual’s Step 5 medications needs of at least 50% with respect to baseline. All analyses will be performed according to the intention-to-treat principle. Baseline characteristics measured on a nominal or ordinal scale will be compared by the chi-square test or analysis of variance, respectively. In the primary analysis, the proportions of patients in the two study groups who successfully reached the primary outcome will be compared with the use of Fisher’s exact test. Subjects will be stratified on the basis of their daily (high or low) steroid dose at the run-in period, and differences between the high-dose and low-dose strata, with regard to the outcome, will be compared by a logistic regression analysis model. An interim analysis will be performed by the O’Brien-Fleming method when 75% of subjects are included into the study to reveal possible significant differences between treatment groups. The daily prednisone dose will be compared by repeated-measures analysis of variance. The number of patients withdrawn from therapy because of adverse reactions or treatment failure will be compared between study groups by Fisher’s exact test. Secondary efficacy variables will be analyzed by the chi-square test and analysis of variance.

## Discussion

Current available medications for chronic severe asthma are not satisfactory and improvement in the management of chronic severe asthma is much needed. It is surprising that the initial encouraging experience of using no- steroidal immunological modifiers for severe asthma has been neglected. Most likely, significant drawbacks in methodology and study design, and disproportionate concerns about immunomodulators’ toxicity have contributed to this. Small sample size, vague disease definition, heterogeneous patient populations, unsatisfactory follow-up period, lack of adequate run-in for stabilization of patients’ steroid requirements, poorly defined response criteria for steroid tapering, short treatment duration and under-dosing have all contributed to the poor quality of earlier trials [22, 23].

However, among the collection of immunologic modifiers, low-dose MTX has a well-established safety record and long-term observational studies have also shown its efficacy and safety in asthma patients [31–33]. In particular, two prospective open-labelled extension studies reported that more than half of the patients treated with MTX for up to 28 months discontinued OCS completely [34, 35]. Furthermore, one double-blind randomized placebo-controlled study (of 46 steroid-dependent asthmatic patients) observed a decrease in corticosteroids in more than half of the patients in the MTX group, showing the drug’s efficacy as a steroid-sparing agent and with good tolerance [24]. The effect of MTX in increasing T-cell’s susceptibility may at least partly account for its OCS-sparing effect in severe asthma [36]. Although no firm conclusion can be reached based on the limited evidence, it is clear that low-dose MTX can be a useful therapeutic option for chronic severe asthma [37]. Large and well-designed controlled studies are now needed to establish a positive role for MTX in chronic severe asthma, and to identify the key characteristics of those who are going to respond to this drug.

The study presented here has been designed to address most of the methodologic problems encountered in earlier clinical trials investigating efficacy and safety of MTX in chronic severe asthma patients. First of all, chronic severe asthma patients will be characterized with great care prior to inclusion; phenotypization of these participants will have to conform to current GINA diagnostic criteria for chronic severe asthma [18], and to the recently established criteria for chronic refractory asthma by the Unbiased Biomarkers for the Prediction of Respiratory Disease Outcome (U-BIOPRED) Consortium [38]. In particular, the inclusion of a run-in period designed to establish patients’ lowest possible GINA Step 5 medications (systemic steroids and/or omalizumab) requirements is very important to confirm the stability and validity of their sub-phenotype.

Secondly, this study will have enough power to endorse the role of add-on MTX as a valuable drug in patients with chronic severe asthma. In estimating the sample size, we had to consider that most clinical trials on patients with chronic severe asthma have shown the futility of using FEV1 as a realistic primary endpoint for this disease phenotype [25–28]. Moreover, patient-reported outcomes such as the AQLQ and ACQ have proved to be acceptable clinical endpoints in mild-to-moderate asthma, but very little is known about their validation in patients with chronic severe asthma. Validation of these important clinical indicators in chronic severe asthma will be possible in the context of our study in a well-characterized cohort of patients with chronic severe asthma. For these reasons, the sample size has been calculated on the basis of a sound and clinically relevant endpoint; no less than a 50% reduction in dosage of Step 5 medications from baseline was considered as a key primary outcome of efficacy for patients with chronic severe asthma. Particular attention has been paid to a clear working definition for the response criteria during steroid and/or omalizumab tapering.

Thirdly, treatment duration and MTX dosing will be sufficient enough to determine if patients with chronic severe asthma will derive beneficial clinical effects (or cumulative adverse events) from regular MTX use. Lastly, this study includes a 12-month duration run-out period, during which modifications of the patients’ asthma symptoms and medications will be carefully documented. This important extension of the study design has been included to crosscheck any potential valuable contribution of low-dose MTX in responders.

Hopefully this study will show substantial reductions in the total dose of Step 5 medications by MTX, thus reducing the risk of serious adverse effects of systemic corticosteroids, and contributing to considerable cost savings. A significant reduction in the total dose of oral steroids will reduce the risk of serious adverse effects such as osteoporosis, diabetes, hypertension and gastrointestinal bleeding. This will lead not only to considerable savings in the form of less frequent monitoring of bone mineral density, fasting glucose and blood pressure, but also through discontinuation of biphosphonates for bone protection, and of proton pump inhibitors for gastrointestinal bleeding prevention. It is worth noting that because of the growing number of patients with chronic severe asthma using omalizumab to improve their disease control, we will end up recruiting not only patients taking regular OCS, but also patients on omalizumab. Therefore, this study has the potential of addressing for the first time the role of low-dose MTX in reducing the total dose of omalizumab without compromising overall disease control. In consideration of the high acquisition cost of omalizumab a significant reduction in the total dose of omalizumab by the much cheaper MTX may lead to substantial savings.

The present study will be the first multicenter randomized controlled trial investigating the role of add-on low-dose MTX in patients with well-phenotyped chronic severe asthma. Hopefully findings from this study will advance current knowledge and recommendations about the characterization, evaluation and treatment of chronic severe asthma [38, 39]. In particular, we expect to confirm that MTX may be considered for inclusion in the current therapeutic arsenal for chronic severe asthma.

## Trial status

We expect to start recruiting patients into the study in September 2015.

## Abbreviations

ACQ: Asthma Control Questionnaire
AQLQ: Asthma Quality of Life Questionnaire
CRO: Contract research organization
CSA: Chronic severe asthma
eCRF: Electronic case report form
FEV1: Forced expiratory volume in 1 second
GCP: Good clinical practices
GINA: Global Initiative for Asthma
LIAF: Lega Italiana Anti Fumo - Anti-Smoking Italian League
MTX: Methotrexate
MTXPGs: MTX polyglutamates
OCS: Oral corticosteroids.
